# Fatal Acute Liver Failure Associated With Presumed Hepatitis B Virus Reactivation During Rituximab Maintenance Therapy: A Case Report

**DOI:** 10.7759/cureus.111434

**Published:** 2026-06-24

**Authors:** James Tran, Calvin Zhou, Asis A Babun, Whinkie Leung, Ali Al-Sarray

**Affiliations:** 1 Academics, Rocky Vista University College of Osteopathic Medicine, Ivins, USA; 2 Military Medicine, Rocky Vista University College of Osteopathic Medicine, Ivins, USA; 3 Internal Medicine, St. Mark's Hospital, Salt Lake City, USA

**Keywords:** acute liver failure, antiviral prophylaxis, case report, hepatitis b reactivation, non-hodgkin lymphoma, rituximab

## Abstract

We report a woman in her 60s with follicular non-Hodgkin lymphoma receiving maintenance rituximab therapy, last administered one month prior to presentation, who developed progressive malaise, jaundice, and acute liver failure. Laboratory evaluation demonstrated severe hepatocellular injury with marked transaminase elevations (alanine aminotransferase: 2,500 U/L; aspartate aminotransferase: 2,400 U/L), hyperbilirubinemia (total bilirubin: 20 mg/dL), and coagulopathy. Hepatitis B virus (HBV) serology demonstrated active infection with elevated hepatitis B surface antigen (HBsAg) and detectable HBV DNA. Baseline HBV screening and antiviral prophylaxis records prior to rituximab initiation were unavailable from the treating institution, limiting the definitive confirmation of pre-existing HBV status. Given the patient's recent rituximab exposure, clinical presentation, and exclusion of alternative etiologies, HBV reactivation was considered the most likely diagnosis. The patient was diagnosed with HBV reactivation associated with rituximab therapy. Despite the initiation of antiviral treatment and aggressive supportive care, her clinical course rapidly deteriorated, complicated by hepatic encephalopathy, acute kidney injury requiring hemodialysis, and hypoxic respiratory failure. She was evaluated for liver transplantation but deemed ineligible due to multiorgan failure and ultimately transitioned to end-of-life care. This case highlights the potentially fatal consequences of HBV reactivation during rituximab therapy and underscores the importance of appropriate screening, prophylaxis, and vigilance in high-risk patients.

## Introduction

Rituximab is a chimeric monoclonal antibody targeting the CD20 antigen on B lymphocytes and is widely used in the treatment of non‑Hodgkin lymphoma and various autoimmune diseases [1]. By inducing prolonged B‑cell depletion, rituximab significantly impairs humoral immunity and predisposes patients to viral reactivation [2]. Patients receiving B‑cell‑depleting therapy represent one of the highest‑risk groups for hepatitis B virus (HBV) reactivation, with reported reactivation rates of 50-64% in the absence of antiviral prophylaxis [3].

HBV reactivation results from the treatment‑induced loss of immune control over latent virus, followed by immune‑mediated hepatocellular injury during immune reconstitution [4]. In immunocompetent adults, approximately 90% of acute HBV infections resolve spontaneously, while the remaining 10% progress to chronic infection [5]. Clinical manifestations of HBV reactivation range from asymptomatic aminotransferase elevation to fulminant hepatitis, cirrhosis, and hepatocellular carcinoma [6]. In immunocompromised patients, HBV reactivation is regarded as a serious complication that may rapidly progress to acute liver failure and multiorgan failure [7]. The American Association for the Study of Liver Diseases (AASLD) defines HBV reactivation as either a ≥100‑fold increase in HBV DNA from baseline or HBV DNA >1,000 IU/mL in patients with previously undetectable levels [8].

Current guidelines from the AASLD, the American Society of Clinical Oncology (ASCO), and the European Conference on Infections in Leukaemia (ECIL‑5) recommend universal HBV screening using hepatitis B surface antigen (HBsAg) and hepatitis B core antibody (anti‑HBc) prior to the initiation of rituximab therapy [9-11]. Patients with evidence of prior HBV infection (HBsAg‑negative/anti‑HBc‑positive) are also recommended to receive antiviral prophylaxis during treatment and for at least 12 months following the completion of therapy [9-11].

Despite these recommendations, severe HBV reactivation continues to occur, most commonly due to incomplete screening or failure to initiate antiviral prophylaxis [12]. In a large retrospective observational study of patients receiving rituximab, although anti‑HBc screening rates improved to 97%, only 23% of anti‑HBc‑positive patients received antiviral prophylaxis, and 87.5% of HBV reactivation cases occurred in patients who did not receive prophylaxis [13]. Antiviral prophylaxis has been shown to reduce the incidence of HBV reactivation during rituximab therapy by as much as 82-87%, underscoring the largely preventable nature of this complication [14].

We report a case of fatal acute liver failure secondary to HBV reactivation in a patient with undocumented baseline HBV serologic status who received maintenance rituximab therapy for non‑Hodgkin lymphoma.

## Case presentation

A Spanish-speaking woman in her 60s was admitted with one week of nausea, vomiting, and progressive jaundice. She reported increasing dark urine and increasing abdominal pain prior to admission. She denies fever or confusion. This occurred on a background of follicular non-Hodgkin lymphoma treated with five cycles of cyclophosphamide, hydroxydaunomycin (doxorubicin), vincristine, and prednisolone (CHOP) chemotherapy with filgrastim support, completed in February 2025.

Following a favorable treatment response, the patient was transitioned to maintenance rituximab therapy, which continued through May 2025, with the final dose administered approximately one month prior to presentation. Three weeks before admission, she developed pharyngitis and completed a course of cephalexin. She denied acetaminophen overuse, alcohol consumption, illicit drug use, use of herbal supplements, or prior history of liver disease. Pre-treatment HBV serological testing results and records regarding antiviral prophylaxis were unavailable from her treating facility in Mexico. The patient demonstrated an excellent response to therapy and was then placed on rituximab maintenance therapy every two weeks, with her most recent dose being administered one month prior to presentation.

On presentation, she was afebrile and hemodynamically stable on room air. She was alert and oriented without focal neurological deficits. Physical examination was notable for scleral icterus and jaundiced skin. The abdomen was soft and non-distended with mild periumbilical tenderness. No stigmata of chronic liver disease were present.

Investigations

On laboratory investigation, the complete blood count was initially unremarkable, with a hemoglobin of 15 g/L, a white cell count of 6.2 K/uL with normal differential, and a platelet count of 145 K/uL. Comprehensive metabolic panel (CMP) demonstrated preserved renal function with creatinine of 0.89 mg/dL, estimated glomerular filtration rate (eGFR) of 73 mL/min/1.73 m^2^, and normal electrolytes. Liver function tests demonstrated severe hepatocellular injury and cholestasis, with alanine transaminase of 2574 U/L, aspartate transaminase of 2395 U/L, total bilirubin of 20 mg/dL (direct bilirubin: 13 mg/dL), and alkaline phosphatase of 188 U/L. Coagulation studies revealed significant synthetic dysfunction with an international normalized ratio (INR) of 2.2 and a prothrombin time of 31.4 s. Serum acetaminophen level was low, and ethanol level was negative. Key laboratory findings on admission and during hospitalization are summarized in Table 1. Serial laboratory assessment demonstrated progressive deterioration despite treatment. Aminotransferase levels remained markedly elevated throughout hospitalization, while total bilirubin increased from 20 mg/dL on admission to a peak of 29.2 mg/dL. Coagulopathy worsened with INR increasing from 2.2 to 6.14. Renal function progressively declined, with creatinine rising from 0.89 mg/dL to 6.92 mg/dL, ultimately necessitating hemodialysis. These findings reflected worsening hepatic synthetic dysfunction and multiorgan failure despite antiviral and supportive therapy.

**Table 1:** Admission and peak laboratory abnormalities during hospitalization Serial laboratory assessment demonstrated progressive clinical deterioration despite treatment. Total bilirubin increased from 20 mg/dL on admission to a peak of 29.2 mg/dL, INR worsened from 2.2 to 6.14, creatinine increased from 0.89 mg/dL to 6.92 mg/dL, and platelet count declined from 145×10³/µL to a nadir of 57×10³/µL. These findings reflected worsening hepatic synthetic dysfunction, acute kidney injury, and progression to multiorgan failure. *Peak transaminase values occurred on admission. ^†^Quantitative HBV DNA values were not consistently available in the medical record; only qualitative evidence of elevated HBV DNA was documented. HBV: hepatitis B virus; HBsAg: hepatitis B surface antigen; ALT: alanine aminotransferase; AST: aspartate aminotransferase; INR: international normalized ratio

Laboratory parameter	Admission (day 0)	Peak/nadir during hospitalization	Reference range
ALT, U/L	2574	2574*	7-56
AST, U/L	2395	2395*	10-40
Total bilirubin, mg/dL	20	29.2	0.2-1.2
Direct bilirubin, mg/dL	13	N/A	0-0.3
Alkaline phosphatase, U/L	188	N/A	44-147
INR	2.2	6.14	0.9-1.1
Creatinine, mg/dL	0.89	6.92	0.6-1.3
Platelet count, ×10³/µL	145	57	150-400
HBsAg	Positive	Positive	Negative
HBV DNA	Elevated†	Elevated†	Undetectable

On imaging evaluation, right upper quadrant ultrasonography was unremarkable, and triphasic computed tomography (CT) of the liver showed normal hepatic morphology and enhancement, a patent portal vein, and mild periportal edema without evidence of biliary obstruction or portal hypertension.

Viral hepatitis testing revealed markedly elevated HBsAg (>1000). Hepatitis C antibody, hepatitis A IgM, and human immunodeficiency virus (HIV) testing were negative. Additional serological and molecular testing for alternative viral and autoimmune causes was unrevealing. HBV DNA was found to be elevated on hospital day 7, though specific quantitative values were not fully characterized in the medical record.

Differential diagnosis

The initial differential diagnosis included drug-induced liver injury, acute viral hepatitis, ischemic hepatitis, autoimmune hepatitis, and hepatic infiltration by lymphoma. Given the lack of abdominal pain or obstructive features along with unremarkable sonography and CT findings, surgical causes of hyperbilirubinemia were considered less likely. Imaging revealed no evidence of cirrhosis and suggested acute hepatic impairment. There was no history of hemodynamic instability to suggest ischemic hepatitis. Although drug-induced liver injury remained a consideration, the patient's history of non-Hodgkin lymphoma treated with maintenance rituximab represented a significant risk factor for HBV reactivation. In the setting of markedly elevated HBsAg and exclusion of alternative etiologies, HBV reactivation was determined to be the primary diagnosis.

Treatment

Rituximab was discontinued, and the patient was started on three-day intravenous N-acetylcysteine on admission. Antiviral therapy with tenofovir 300 mg daily was started on hospital day 4. Supportive care, including pain management, nutritional support through a nasogastric tube, and lactulose, was initiated on day 8 following acute deterioration in status. She was later transitioned to entecavir 0.5 mg post-dialysis on day 10 for concerns of nephrotoxicity.

Outcome and follow-up

Despite therapy, the patient's liver function continued to deteriorate as she developed severe hepatic encephalopathy, with her total bilirubin as 29.2 and INR of 6.14. She also developed renal failure, suspected to be due to a constellation of hepatorenal syndrome alongside tenofovir and contrast administration, with a creatinine peak of 6.92 and an eGFR of 3 mL/min/1.73 m^2^ on day 12 of admission, which was managed with daily hemodialysis beginning on hospital day 8. Her course was further complicated by hypoxic respiratory failure requiring bilevel positive airway pressure (BiPAP) and presumed hospital-acquired pneumonia treated with broad-spectrum antibiotics. She was evaluated for liver transplantation but deemed not a candidate due to multiorgan failure. After goals-of-care discussions, the family elected to return to Mexico for continued dialysis and end-of-life care. She was discharged in critical condition. Future follow-up confirmed the patient passed in transit.

The clinical timeline is presented in Table 2.

**Table 2:** Clinical timeline of disease progression

Timepoint	Clinical event
-24 months	Diagnosed with follicular non‑Hodgkin lymphoma; lymph node excision and induction chemotherapy initiated
-10 months	Completion of induction chemotherapy; maintenance rituximab initiated
-1 month	Last dose of rituximab administered
-2 weeks	Pharyngitis treated with antibiotics; progressive malaise developed
-3 days	Onset of nausea and abdominal discomfort
Hospital day 0	Admission with jaundice, nausea, and vomiting; severe hepatocellular injury identified
Hospital day 1	Gastroenterology consultation; findings consistent with hepatitis B virus reactivation
Hospital day 4	Antiviral therapy initiated
Hospital days 5-7	Progressive acute kidney injury and worsening liver failure
Hospital day 8	Critical decompensation with hepatic encephalopathy; hemodialysis initiated
Hospital day 10	Multiorgan failure; palliative care consultation
Hospital days 11-12	Respiratory failure; discharged to pursue end‑of‑life care

## Discussion

HBV reactivation during rituximab therapy is a well‑recognized complication with a predictable clinical course, yet it continues to result in catastrophic outcomes [11]. This case of fatal acute liver failure occurring during maintenance rituximab therapy highlights the limitations of current HBV screening strategies and the false reassurance that negative or undocumented serological testing may provide in real‑world clinical practice. It underscores how a predictable risk can still translate into irreversible disease when latent infection goes unrecognized and opportunities for prevention are missed.

Although the biological mechanisms underlying HBV reactivation during rituximab therapy are well-established, their clinical implications are often underappreciated [11]. Reactivation typically evolves silently, with unchecked viral replication occurring during periods of profound immunosuppression. Clinical hepatitis often emerges later, when immune recovery triggers immune‑mediated hepatocyte injury [12]. As a result, patients frequently present at an advanced stage of disease, when jaundice, coagulopathy, and hepatic encephalopathy have already developed and therapeutic options are limited [12]. This delayed and abrupt presentation helps explain why HBV reactivation, despite its predictable natural history, continues to result in severe liver failure and high mortality in contemporary clinical practice [11].

A major limitation of this case is the absence of documented baseline HBV serological testing and antiviral prophylaxis records prior to the initiation of immunosuppressive therapy. Consequently, definitive confirmation of HBV reactivation according to standard guideline definitions is not possible. The patient's HBV infection may have represented reactivation of previously resolved or occult infection, chronic HBV infection that was not recognized before treatment, or, less likely, a newly acquired infection. Nevertheless, the temporal association with rituximab therapy, elevated HBV markers, exclusion of alternative etiologies, and characteristic clinical course strongly supported HBV reactivation as the most likely diagnosis. Anti‑HBc immunoglobulin G antibodies may become detectable as early as six weeks following acute infection, introducing uncertainty regarding the timing of this patient's HBV exposure [13]. Infection may therefore have occurred prior to the initiation of chemotherapy, been acquired during chemotherapy with subsequent resolution and transition to a latent state, or occurred shortly before clinical presentation. Although the patient was reportedly adherent to prescribed oncologic therapies, baseline HBV screening results could not be verified.

In Mexico, oncologic practice commonly follows international guidelines issued by organizations such as the National Comprehensive Cancer Network (NCCN), the European Society for Medical Oncology (ESMO), and the ASCO, all of which recommend universal HBV screening with HBsAg and anti‑HBc prior to the initiation of anti‑CD20 monoclonal antibody therapy [5,12,13]. These guidelines also recommend antiviral prophylaxis for patients at risk of HBV reactivation, continued for up to 12 months following the completion of therapy [5,12,13]. However, even when such screening is performed, reliance on serological testing alone may fail to identify all patients at risk. Notably, current guidelines do not recommend routine repeat screening after chemotherapy initiation.

False‑negative HBV serological testing may occur through several mechanisms and has important implications for the management of patients receiving rituximab. Mutations in the HBV surface (S) gene can alter the antigenic conformation of HBsAg, rendering it undetectable by standard immunoassays [14]. Such escape mutations may arise spontaneously or be masked by circulating immune complexes [14]. In addition, approximately 20% of patients with occult HBV infection lack detectable anti‑HBc antibodies, likely due to waning antibody titers or impaired humoral immune responses [15]. Consequently, reliance on HBsAg and anti‑HBc testing alone may fail to identify a subset of patients with latent infection who remain at risk for reactivation during profound immunosuppression.

Despite these limitations, anti‑HBc remains the most practical and reliable serologic marker for occult HBV infection in routine clinical practice, given the relative insensitivity of standard HBsAg assays and the impracticality of liver biopsy for screening purposes [16]. Moreover, low‑level viremia below standard assay detection thresholds may escape identification at baseline yet expand rapidly under immunosuppressive conditions. Collectively, these factors highlight how patients at highest risk for HBV reactivation may paradoxically be those in whom standard screening performs least reliably.

These considerations suggest that HBV screening in high‑risk patients may benefit from a longitudinal rather than a single timepoint approach. Repeat testing for HBsAg and anti‑HBc during prolonged or maintenance immunosuppression may help identify patients whose serologic status evolves over time due to immune reconstitution, waning antibody levels, or increasing viral replication. However, rituximab‑induced impairment of humoral immunity can result in declining or undetectable antibody titers, potentially leading to false‑negative anti‑HBc or anti‑HBs results even in patients with prior exposure [17].

In patients who remain seronegative despite ongoing high‑risk therapy, adjunctive HBV DNA testing may further improve risk stratification by detecting occult viral replication independent of host immune responses. This approach may enable earlier initiation of antiviral prophylaxis before immune‑mediated liver injury occurs. Ultimately, this case underscores that HBV reactivation during rituximab therapy remains a clinically significant and potentially fatal complication not because it is unpredictable, but because current prevention strategies may fail in profoundly immunosuppressed patients. Once clinical hepatitis develops, therapeutic options are limited, and outcomes remain poor.

## Conclusions

This case highlights a fatal episode of presumed HBV reactivation occurring during maintenance rituximab therapy. Although baseline HBV serologic status could not be confirmed, the clinical presentation was highly suggestive of HBV reactivation resulting in acute liver failure and multiorgan dysfunction. The case underscores the importance of comprehensive HBV screening, appropriate antiviral prophylaxis, and continued vigilance in patients receiving prolonged B-cell-depleting therapy. Further investigation is warranted to determine whether repeat HBV surveillance during long-term immunosuppression may improve the identification of patients at risk for delayed reactivation.
